# Detection of Cardiopulmonary Activity and Related Abnormal Events Using Microsoft Kinect Sensor

**DOI:** 10.3390/s18030920

**Published:** 2018-03-20

**Authors:** Ali Al-Naji, Javaan Chahl

**Affiliations:** 1 School of Engineering, University of South Australia, Mawson Lakes, SA 5095, Australia; 2 Electrical Engineering Technical College, Middle Technical University, Al Doura 10022, Baghdad, Iraq; 3 Joint and Operations Analysis Division, Defence Science and Technology Group, Melbourne, VIC 3207, Australia; Javaan.Chahl@unisa.edu.au

**Keywords:** cardiopulmonary signal, video magnification techniques, improved signal decomposition technique, blind source separation, canonical correlation analysis, frame subtraction method

## Abstract

Monitoring of cardiopulmonary activity is a challenge when attempted under adverse conditions, including different sleeping postures, environmental settings, and an unclear region of interest (ROI). This study proposes an efficient remote imaging system based on a Microsoft Kinect v2 sensor for the observation of cardiopulmonary-signal-and-detection-related abnormal cardiopulmonary events (e.g., tachycardia, bradycardia, tachypnea, bradypnea, and central apnoea) in many possible sleeping postures within varying environmental settings including in total darkness and whether the subject is covered by a blanket or not. The proposed system extracts the signal from the abdominal-thoracic region where cardiopulmonary activity is most pronounced, using a real-time image sequence captured by Kinect v2 sensor. The proposed system shows promising results in any sleep posture, regardless of illumination conditions and unclear ROI even in the presence of a blanket, whilst being reliable, safe, and cost-effective.

## 1. Introduction

The Kinect is a motion-sensing technology developed by Microsoft for gaming purposes with the XBOX360 gaming console. The first version of Kinect sensors (Kinect v1) was released in 2010 based on structured light coding technology [1]. Later, Microsoft developed it to be compatible with Windows using a standard development kit (SDK) and a power conversion adaptor. The next version of the Kinect sensors (Kinect v2) was released in 2014 based on a time of flight (ToF) technology [2] with improved specifications compared to the original Kinect in terms of regarding to speed, accuracy, and increased field of view [3,4,5,6,7]. The built-in Kinect software library can provide many processing techniques, including body tracking, three-dimensional body reconstruction, face detection, joint tracking, skeletal tracking, and human recognition using the image sequences acquired from three built-in optical sensors: a red, green, and blue (RGB) sensor, infrared (IR) sensor, and depth sensor. A comparison between the Microsoft Kinect v1 and Kinect v2 is shown in Table 1.

**Figure 1:**
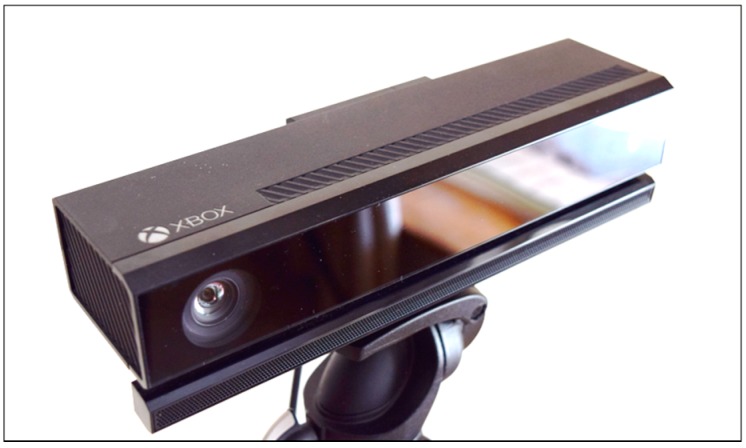
Microsoft Kinect v2 sensor.

In addition to the specifications listed in Table 1, the Kinect v2 sensor is very robust regarding body rotation, flip, scale changes, illumination changes, cluttered backgrounds, and distortions as well as being commercially available at low cost and with high-level programming interfaces, making it a promising technology for many clinical and biomedical imaging applications. The external view of the Microsoft Kinect v2 sensor is shown in Figure 1.

In recent years, an increasing number of research projects have used the Microsoft Kinect sensor as a remote device to detect the cardiopulmonary activity in diagnostic and healthcare applications. For instance, a study by Xia and Siochi [8] proposed a Kinect-based respiratory monitoring system to track respiratory motion in real-time by averaging depth values for pixels in the thoracic region. The region of interest (ROI) in their study was manually selected by the user through placing a translation surface on the patient’s thorax in the Kinect field of view. Therefore, their study needs the patient to stay in a stationary condition to obtain clear ROI for measurement. Another study by Smilkstein et al. [9] demonstrated that it is possible to extract the cardiac signal based on processing of the RGB images obtained from a Kinect v1 sensor when the facial skin colour was first magnified by the Eulerian video magnification (EVM) technique [10]. However, their experiment was run in a completely static environment and suffered from system failure when the subject moved. A study by Bernacchia et al. [11] proposed a remote monitoring system based on a Kinect v1 sensor to extract cardiopulmonary signal at three ROIs (neck, thorax, and abdomen), where all of the regions were manually selected. Remote sleep monitoring systems have been proposed in some studies [12,13] to detect thorax movement based on the Kinect v1 sensor. These studies used the information acquired from the depth sensor to track patient’s thorax over time during sleep. However, they used the centre of the image as an effective ROI without a tracking system that would lead to distorting and biasing of the results when any unexpected movement exists. Other studies [14,15] detected the respiration peaks for the patient while facing the Kinect v1 sensor based on depth map information. However, unclear ROI and subject movement were the main limitations. A study by Aoki et al. [16] proposed a remote respiration measurement system using the Kinect v1 sensor to extract respiratory activity for a patient undergoing an exercise tolerance test. Detection of respiratory activity and some sleep disorders based on the Kinect sensor has been investigated by Centonze et al. [17]. That study was also prone to some limitations, including unclear ROI, noise artefacts, and subject movement. Another study by Yang et al. [18] estimated the cardiac signal from head motion tracking at the nasal tip region using the Kinect v2 depth sensor. However, their study was prone to motion artefacts and noise due to a low bit-depth representation captured by the Kinect depth sensor and subject movement. To address subject movement, Harte et al. [19] developed a remote monitoring system for capturing dynamic thoracic wall motion using four Kinect sensors by creating a 3D time-varying view of the subject’s torso. Although their proposed system may have a benefit in non-stationary scenarios, some errors may be generated in the 3D reconstruction when the sensors were unsynchronized in time and frequency. A study by Tahavori et al. [20] assessed how the Kinect depth sensor might be used to solve the problems associated with patient setup misalignment and respiratory motion management that may be a significant source of error in radiotherapy. Kumagai et al. [21] proposed a non-contact motion tracking system to detect respiratory signals at multiple points on the abdominal-thoracic region based on the Kinect depth sensor. However, there was only one accurate position, which was when a subject faces the Kinect sensor. Lee et al. [22] presented a Kinect-based sleep monitoring system to track whole human body joints and detecting of sleep patterns and postures using a Kinect v2 sensor. The human body in that study could not be covered with a blanket. Another study by Gambi et al. [23] also used an EVM technique to reveal facial skin colour changes from RGB frames obtained by the Kinect sensor, when only the face region was exposed for analysis. Estimating the cardiac signal and rhythm at different head poses using 3D head motion tracking based on a Kinect depth sensor was proposed by Yang et al. [24]. The limitation was the difficulty of extracting the cardiac signal when the subject was lying down on a bed and some other problems caused by unclear ROI. Recently, we proposed a real-time monitoring system to extract the respiratory activity and to detect apnoea for children using the Kinect v2 sensor in any given sleep posture and any environmental setting [25]. In the current study, a real-time monitoring system based on the Kinect v2 sensor was proposed to extract the heart rate (HR) and respiratory rate (RR) and differentiate the various forms of related abnormal cardiopulmonary events (e.g., tachycardia, bradycardia, tachypnea, bradypnea, and central apnoea).

This paper is organized as follows: Section 1 presents the description of the Microsoft Kinect sensor and introduces related work in the biomedical field. Section 2 describes the relevance of cardiopulmonary activity and related abnormal cardiopulmonary events. Section 3 presents the methods and procedures of the proposed system, including the participants, experimental setup, validation methods, system framework, and data analysis. The experimental results of the proposed system with different environmental settings are presented and discussed in Section 4 and Section 5, respectively. Finally, concluding remarks are outlined in Section 6.

## 2. Cardiopulmonary Activity and Related Abnormal Events

Cardiopulmonary activity causes volumetric changes resulting from the heart muscle and main respiratory muscle (diaphragm), which can provide useful information to extract the cardiopulmonary signal. Volumetric changes in the thorax resulting from the heart muscle are between 0.2 and 0.5 mm and falling within a frequency band of 1 Hz and 2 Hz, while the changes resulting from the diaphragm are between 4 and 12 mm and falling within a frequency band of 0.1 Hz and 0.3 Hz [26,27]. The HR and RR are the main physiological signs within the cardiopulmonary activity and are often indicators of abnormal cardiopulmonary conditions, including tachycardia (when the HR exceeds the normal range), bradycardia (when the HR is under the normal range) [28], tachypnea (when the RR exceeds the normal range), bradypnea (when the RR is under the normal range), and central apnoea (when there is no breathing) [29]. The normal range of physiological signs (HR and RR) is shown in Table 2.

## 3. Methods & Procedures

### 3.1. Participants

The research presented in this study was conducted using 10 participants (five males and five females) within the ages of 1–6 years and one adult (36 years) who participated to simulate abnormal cardiopulmonary events. The video data was captured using a Microsoft Kinect v2 sensor with a resolution of 1920 × 1080 and a frame rate of 30 fps. The Kinect was connected to a laptop with the SDK installed and a conversion power adaptor. The Ethical model was granted by the University of South Australia Human Research Ethics Committee (Adelaide, South Australia, Protocol number: 0000034901). A written informed consent was obtained from participants’ parents after a full explanation of the research procedure before commencing the experiment.

### 3.2. Experimental Setup & Validation Methods

The Kinect v2 sensor was installed in a home environment oriented at 45° and at a distance of 2.5–3 m from the participant’s thorax. The experiment was for approximately 1–3 h for each participant and repeated at different times of day and different environmental settings to obtain sufficient video data. The experimental results obtained from the participants at many postures with and without a blanket were set in two environmental settings. In the first setting, the experiments were implemented in a well-lit environment (~500 lux) using video data obtained from the RGB sensor. In the second setting, the experiments were implemented in a dark environment (<1 lux) using video data obtained from the IR sensor. The validation procedure was carried out using two reference instruments: a finger pulse oximeter (Rossmax SA210, accuracy ±1 digit) and Piezo respiratory belt transducer MLT1132 (http://www.adinstruments.com/products/mlt1132).

### 3.3. System Framework & Data Analysis

**Figure 2:**
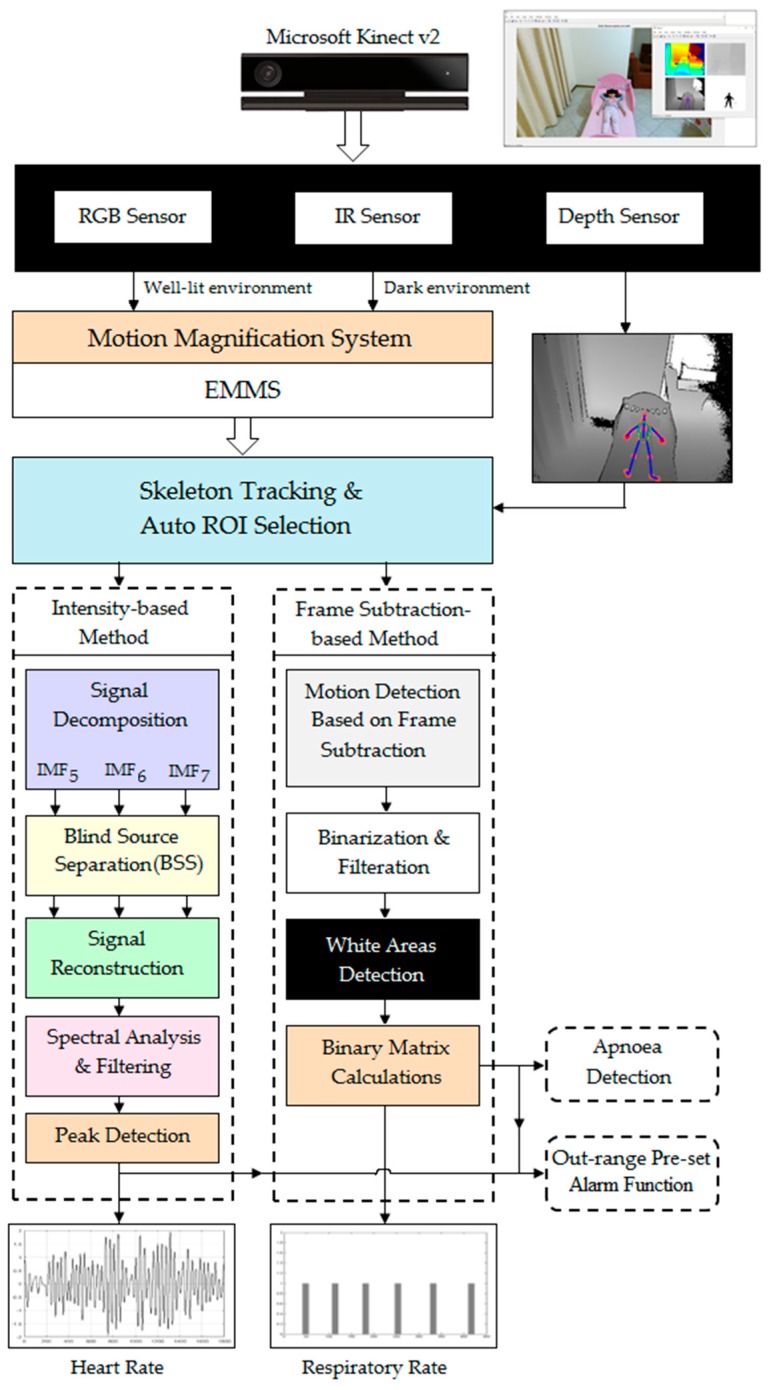
Block diagram of the proposed system based on a Microsoft Kinect v2 sensor.

The framework of the proposed system to extract cardiopulmonary signal and detect abnormal cardiopulmonary events from video data captured by a Microsoft Kinect v2 sensor is presented in Figure 2.

The magnification technique proposed in [30] was used to provide a real-time magnification since this technique has better noise performance and video quality than other magnification techniques. The magnification process was used to either magnify the video data captured by the RGB sensor in a well-lit environment or to magnify the video data captured by the IR sensor in a dark environment, since the RGB sensor is not effective in the dark.

**Figure 3:**
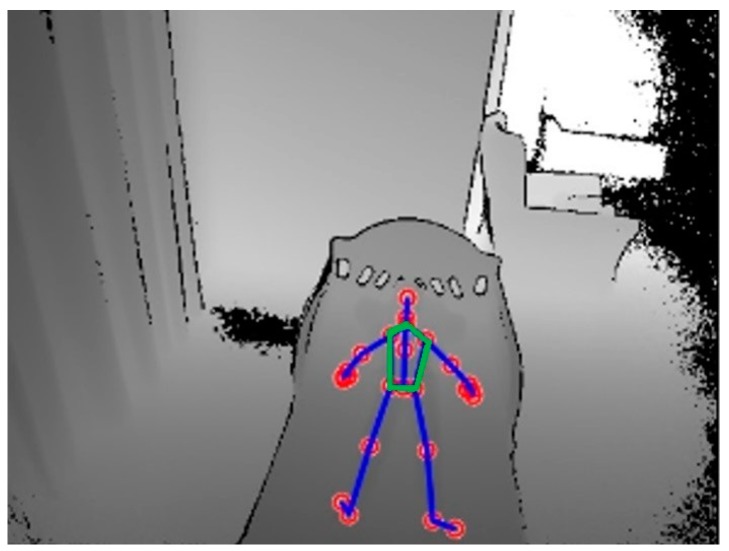
Skeletal joints of the child (5 years) provided from the Kinect code library and the selected region of interest (ROI; the green pentagon defined by the 5 joints).

To select the effective ROI, where the cardiopulmonary signal is significant, the depth sensor was used to detect and track the movement of the abdominal-thoracic region caused by cardiopulmonary activity. Kinect v2 sensor can efficiently detect movement of the human body within an operative measuring range of 0.5–4.5 m using the depth information by tracking the positions of 25 skeletal joints. Using the Kinect code library, we selected five joints corresponding to the abdominal-thoracic region that are located within the left and right shoulder joints, left and right hip joints, and spine shoulder joint as shown in Figure 3.

Once the ROI was selected, the proposed system divides into two main processing methods: the intensity-based method introduced in [31], which was used to extract the cardiac signal and detect tachycardia and bradycardia, and the frame-subtraction-based method introduced in [32], which was used to extract the respiratory signal and to detect tachypnea, bradypnea, and central apnoea.

#### 3.3.1. Extraction of Cardiac Signal Using Intensity-Based Method

The mean intensity of the image pixel values within the selected ROI was calculated for each frame to obtain a time-series signal. The time-series signal was further preprocessed by applying a 2nd order Butterworth band-pass filter with the selected frequencies 0.75–3 Hz corresponding to 45–180 beats/min and remove low-frequency movements such as respiration and changes in posture from the signal.

**Figure 4:**
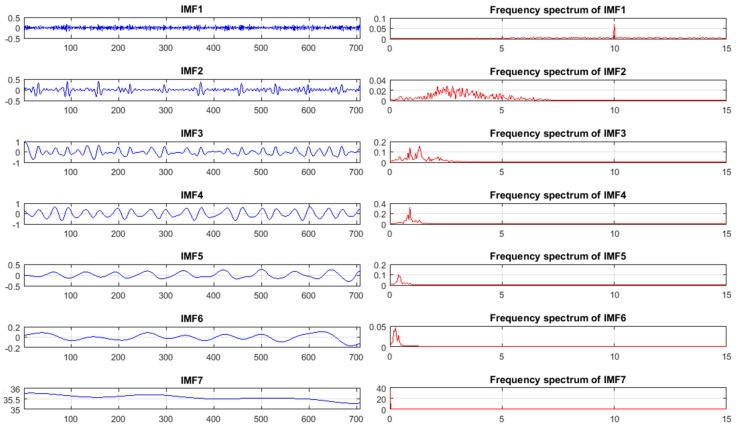
Intrinsic mode function (IMF) components of the improved complete ensemble empirical mode decomposition (CEEMD) technique with noise adaptation and their frequency spectrums.

To remove noise and motion artefacts, the acquired signal was decomposed into seven intrinsic mode function (IMF) components with different amplitudes and frequencies based on an improved complete ensemble empirical mode decomposition (CEEMD) technique with noise adaptation [33]. This technique decomposes the signal of interest into a set of amplitude and frequency components, called intrinsic mode functions (IMFs), with higher efficiency, less noise, and more physical meaning than other decomposition techniques such as empirical mode decomposition (EMD) [34], ensemble EMD [35], and complete ensemble EMD [36]. In addition, this improved technique outperforms other signal decomposition techniques in terms of reduction of the amount of residual noise from the modes and spurious modes overlapping [37]. The IMF components of the improved CEEMD technique with noise adaptation and their frequency spectra are shown in Figure 4. 

**Figure 5:**
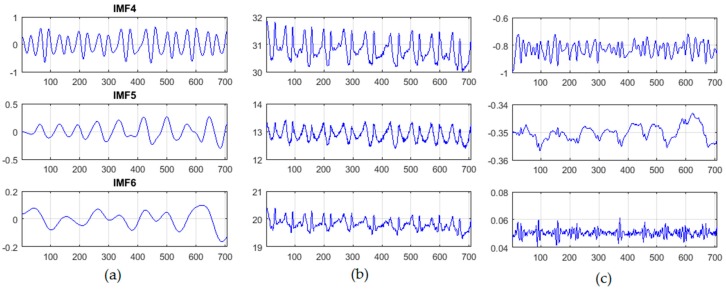
The canonical correlation analysis (CCA) technique (**a**) The selected IMF components (S) (**b**) Transformation [*x* = WS], where W is an un-mixing matrix and (**c**) CCA outputs [*y* = CCA (*x*)].

The decomposition process was then followed by blind source separation (BSS) based on canonical correlation analysis (CCA) technique [38]. The CCA is an effective signal processing technique that can be used as a BSS to separate a number of mixed signals [38,39] and remove noise artefacts from biomedical signals [40,41,42,43]. The CCA can also achieve better performance for the BSS than can independent component analysis (ICA), and has less computational complexity than does ICA [44,45,46]. Once the IMF components were extracted, components with the best cardiac frequency spectra were then selected as inputs to the CCA technique as shown in Figure 5.

The output signal from the CCA technique that has the most resemblance to the expected cardiac signal was then selected. A frequency spectrum analysis based on the fast Fourier transform (FFT) informs the estimation of heart pulse, followed by the MATLAB built-in peak detection method to calculate the pulse beats per minute.

As the normal range of HR for the selected age group (1–6 years) is between 70 and 150 beats/min (See Table 2), a pre-set alarm function is proposed with the system when the HR results fall outside this range, thus detecting abnormal cardiac events for children (e.g., tachycardia and bradycardia). Because the pre-set alarm system relies on age of the participants, it will use a different HR range (60–100 beats/min) when applied to an adult participant.

#### 3.3.2. Extraction of Respiratory Signal Using a Frame-Subtraction-Based Method

Motion in the abdominal-thoracic region resulting from the respiration was detected using the frame subtraction method to recognise the presence of breathing in the consecutive frame sequence as follows [47]:

(1) $$\left|I_{t}\left(i,j\right)-I_{t-1}\left(i,j\right)\right|\geq \tau$$

where $I_{t}\left(i,j\right)$ and $I_{t-1}\left(i,j\right)$ are the intensities of the current and previous images, respectively, and τ is a threshold (0–255) to describe the intensity change. Motion caused by respiratory movement occurs when the difference is greater or equal to τ. Thresholds at ≥110 were set to generate binary images. After determining the motion above threshold τ in the selected ROI caused by respiration, binarization processing methods, including a contrast-limited adaptive histogram equalization [48] and Morphological filtering, were used to enhance the local contrast of image sequences and remove noise from the image sequences. A new measurement approach was proposed to convert image sequences into a binary matrix. The binary matrix was then converted into a binary raw vector to deal with 0 and 1, where 0 represents the dark area (no motion in the image) and 1 represents the white area (a motion in the image). Let $A_{i}$ be a binary vector of length N for a number of consecutive breaths.

(2) $$A_{i}=\;\left[A_{i}\left(1\right),\;A_{i}\left(2\right),\;A_{i}\left(3\right),\;\ldots ,\;A_{i}\left(N\right)\right]$$

where $A_{i}$ is a binary vector that contains values 0 and 1. The number of zeros ($z_{N}$) and number of ones ($o_{N}$) in $A_{i}$ can be calculated as follows [25]:

(3) $$z_{N}=\;N-\;\sum_{i=1}^{N}A_{i}$$

(4) $$o_{N}=\;N-z_{N}.$$

To determine differences between adjacent elements of $A_{i}$, Let $B=diff\left([0\;A_{i}\right])$ return a vector of length N−1. The elements of B are the differences between adjacent elements of $A_{i}$ as follows

(5) $$B\;=\;\left[A_{i}\left(2\right)-A_{i}\left(1\right),\;A_{i}\left(3\right)-A_{i}\left(2\right),\;...,\;A_{i}\left(N\right)-A_{i}\left(N-1\right)\right]>0.$$

To determine the positions of nonzero values in B, a function C=find(B) was applied to return a vector of length M, containing nonzero values as follows:

(6) $$C\;=\;\left[C_{1},\;C_{2},\;C_{3},\;\ldots ,\;C_{M}\right].$$

By calculating the differences in Equation (6) and multiplying them by the frame interval of Kinect sensor (1/30 fps = 0.0334 s), the vector of respiratory cycles $\left(R_{c}\right)$ in a time (t) can be measured as follows:

(7) $$R_{c}\;=\;\left[Rc_{1}\;Rc_{2},\;\ldots ,\;Rc_{M-1}\right]\;\times \;0.0334.$$

Now, the measured value $M_{v}$ of the respiratory signal per minute can be calculated as follows:

(8) $$M_{v}=\;\frac{60\;s}{Rc}.$$

As the normal range of RR for the selected age group is between 16 and 30 breaths/min, a pre-set alarm function was also considered with the proposed system when the RR results fall outside this range and thus detecting some related abnormal breathing events (e.g., bradypnoea and tachypnoea).

#### 3.3.3. Sleep Apnoea Detection

Central apnoea is a cessation of respiration for 20 s or more or less, due to a short withdrawal of the central nervous system signal to the muscles responsible for respiration [49]. This study describes a new system which can detect central apnoea for people with compromised respiratory reflex.

The vector from Equation (7) was stored in the MATLAB workspace and the previous steps were repeated for the next round to obtain further respiratory cycles. Now, to detect apnoea in the respiratory signal, the following relations were used:

(9) $$Rc_{current}\;=Rc_{2}-Rc_{1}=\;\{\begin{array}{c} Rc_{current}\approx \;Rc_{1},\;Rc_{2},\;\ldots ,\;Rc_{M-1}\;\Rightarrow normal\\ Rc_{current}\;\geq \;10\;s\Rightarrow Apnea \end{array}$$

where any cessation between two consecutive respiratory cycles of more than 10 s was detected as apnoea.

## 4. Experimental Results

This section presents experimental results from evaluating the performance of the proposed remote imaging system against the measurements obtained from the reference instruments in two environmental settings (well-lit environments and dark environments). The analysis examines the performance of the system for participants lying down on a bed with and without a blanket. The limits of agreement, level of correlation, and error rate were calculated for the proposed system using the Bland–Altman method [50], Pearson’s correlation coefficient (PCC), Spearman correlation coefficient (SCC), Kendall correlation coefficient (KCC), root mean square error (RMSE), and mean absolute error (MAE).

### 4.1. Measurements of Cardiac Activity

**Figure 6:**
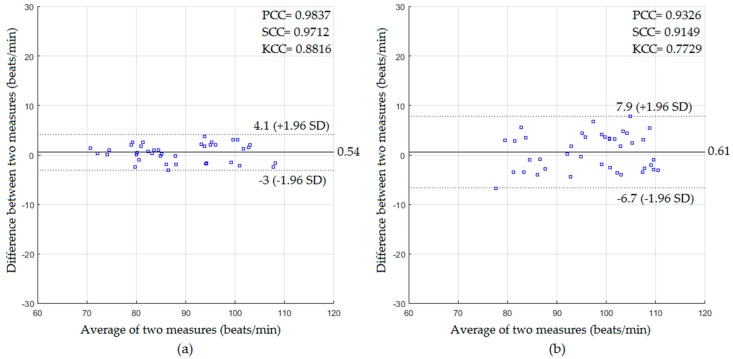
The statistics of HR measurements obtained in a well-lit environment for participants (**a**) without a blanket; (**b**) with a blanket.

In a well-lit environment, the performance of the proposed system of HR measurement was investigated when the video data obtained from the Kinect RGB sensor was used. The cardiac signal was extracted using the intensity-based method. The statistics for the video data captured with and without a blanket based on the Bland–Altman method are presented in Figure 6.

Using the data captured for the participants without a blanket as shown in Figure 6a, the mean bias, lower and upper limit of agreement between the predicted HR and the measured HR were 0.54, −3, and +4.1 beats/min with correlation coefficients of 0.9837, 0.9712, 0.8816 for the PCC, SCC, and KCC, respectively, and error rates of 1.88 beats/min and 1.64 beats/min for the RMSE and MAE, respectively. For the data with a blanket as shown in Figure 6b, the mean bias, lower and upper limit of agreement were 0.61, −6.7, and +7.9 beats/min with PCC of 0.9326, SCC of 0.9149, KCC of 0.7729, RMSE of 3.71 beats/min, and MAE of 3.33 beats/min.

**Figure 7:**
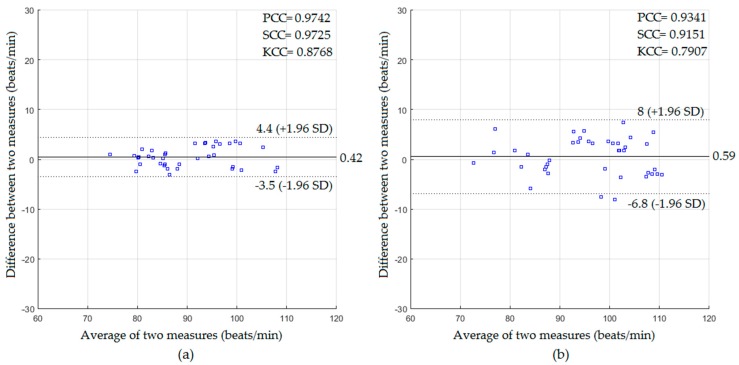
The statistics of HR measurements obtained in a dark environment for participants (**a**) without a blanket; (**b**) with a blanket.

The experiment was repeated under dark environmental conditions. The cardiac signal was extracted in this setting using the video data obtained from the Kinect IR sensor based on the intensity-based method. The statistical results with and without a blanket using the Bland–Altman method are presented in Figure 7.

Using the data captured for the participants without a blanket as shown in Figure 7a, the mean bias, lower and upper limit were 0.42, −3.5, and +4.4 beats/min with PCC of 0.9742, SCC of 0.9725, KCC of 0.8768, RMSE of 2.03 beats/min, and MAE of 1.73 beats/min. For the data with a blanket as shown in Figure 7b, the mean bias, lower and upper limit were 0.59, −6.8, and +8 beats/min with PCC of 0.9341, SCC of 0.9151, KCC of 0.7907, RMSE of 3.78 beats/min, and MAE of 3.28 beats/min.

Because the subjects in this study were healthy participants of a young age, an adult participant (age of 36 years) was instructed to partake in three scenarios before videoing at both light and dark environmental settings to create situations of cardiac events. The first scenario (S1) includes an easy exercise on a treadmill for 5 min. The second scenario (S2) includes a hard exercise on a treadmill for 30 min. The participant for both scenarios was then asked to lie down on a bed with and without a blanket for videoing. The third scenario (S3) includes resting on a bed (sleep) with and without a blanket. Table 3 shows different situations of cardiac events for adult participant at two environmental settings.

It is clear from Table 3 that the proposed system could detect normal readings for HR in both environmental settings with and without a blanket for S1. As the normal range of HR for adult is 60–100 beats/min, the proposed system could also detect abnormal cardiac events that fall outside this range in both environmental settings and even in the presence of a blanket for S2 and S3.

### 4.2. Measurements of Respiratory Activity

**Figure 8:**
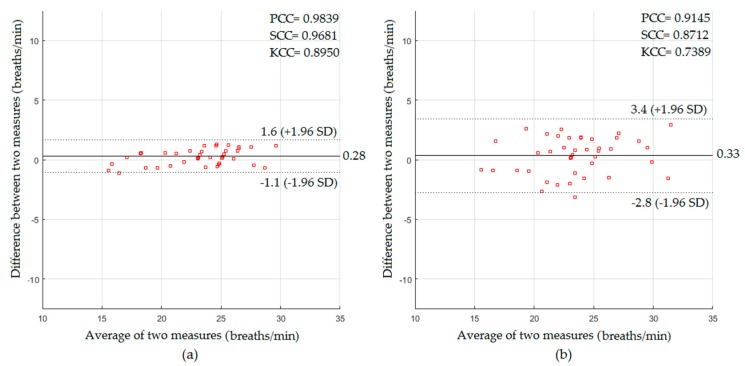
The statistics of RR measurements obtained in a well-lit environment for participants (**a**) without a blanket; (**b**) with a blanket.

In a well-lit environment, the performance of the proposed system of RR measurement was investigated when video data obtained from the Kinect RGB sensor was used. The respiratory signal was extracted using the frame-subtraction-based method. The statistical results for the participants with and without a blanket based on the Bland–Altman method are presented in Figure 8.

The Bland–Altman plot for the RR measurements from the participants without a blanket (see Figure 8a) showed a mean bias of 0.28 breaths/min with a lower limit of −1.1 breaths/min and an upper limit of +1.6 breaths/min, with correlation coefficients of 0.9839, 0.9681, and 0.895 of PCC, SCC, and KCC respectively, and error rates of 0.74 breaths/min of RMSE and 0.64 breaths/min of MAE, whereas the Bland–Altman plot for the RR measurements from the participants with a blanket (see Figure 8b) led to 0.33 breaths/min of mean bias, −2.8 to +3.4 breaths/min of limits of agreement, 0.9145 of PCC, 0.8712 of SCC, 0.7389 of KCC, 1.59 breaths/min of RMSE, and 1.39 breaths/min of MAE.

**Figure 9:**
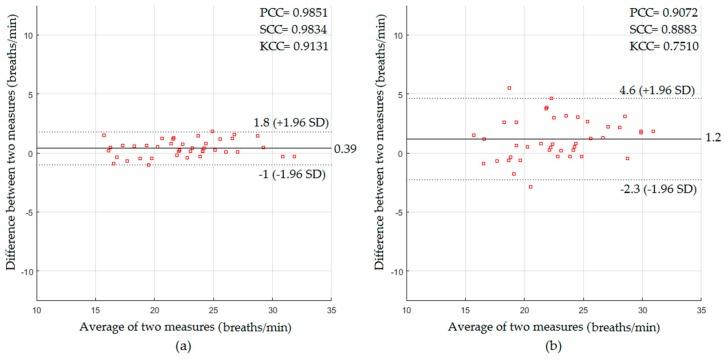
The statistics of RR measurements obtained in a dark environment for participants (**a**) without a blanket; (**b**) with a blanket.

In a dark environment, the respiratory signal was extracted using video data obtained from the Kinect IR sensor based on the frame-subtraction-based method. The statistical results with and without a blanket based on the Bland–Altman method are presented in Figure 9.

The Bland–Altman plot for the RR measurements from the participants without a blanket (see Figure 9a) showed a mean bias of 0.39 breaths/min with a lower limit of −1 breaths/min and an upper limit of +1.8 breaths/min, with PCC of 0.9851, SCC of 0.9834, KCC of 0.9131, RMSE of 0.81 breaths/min, and MAE of 0.66 breaths/min, whereas the Bland–Altman plot for the RR measurements from the participants with a blanket (see Figure 9b) showed a mean bias of 1.2 breaths/min with a lower limit of −2.3 breaths/min and an upper limit of +4.6 breaths/min, with PCC of 0.9072, SCC of 0.8883, KCC of 0.751, RMSE of 2.1 breaths/min, and MAE of 1.63 breaths/min.

Also, we asked an adult participant (36 years) to create a simulation for abnormal respiratory events at two environmental settings by asking him to do three scenarios with and without a blanket. The first scenario (S1) is to breathe normally for one minute. The second scenario (S2) is to breathe more than 30 times for one minute. The third scenario (S3) is to breathe less than 12 times for one minute. For adult participant, tachypnea occurs when the RR is more than 20 breaths/min, while bradypnea occurs when the RR is less than 12 breaths/min. Table 4 shows different situations of respiratory events at two environmental settings.

It is clear from Table 4 that the proposed system could recognize the abnormal respiratory events that fall outside the normal RR range in both environmental settings with and without a blanket.

### 4.3. Apnoea Detection

**Figure 10:**
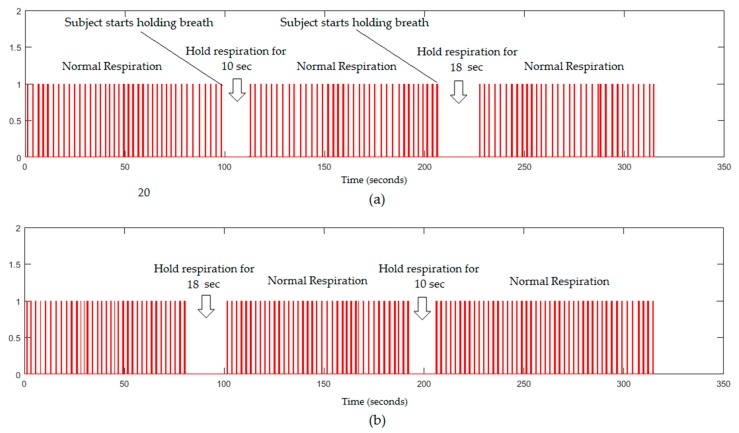
The respiratory signals (5 min) obtained from the proposed system in a well-lit environment, (**a**) without a blanket; (**b**) with a blanket.

In this section, the performance of the proposed system to detect sleep apnoea was investigated at two environmental settings. The respiratory signal acquired by the frame-subtraction-based method might be used to detect the apnoea during sleep. Because all participants were apparently healthy, an adult had been asked to hold his breath twice during videoing to create a similar situation to apnoea. The respiratory signal obtained from the proposed system in a well-lit environment is shown in Figure 10.

**Figure 11:**
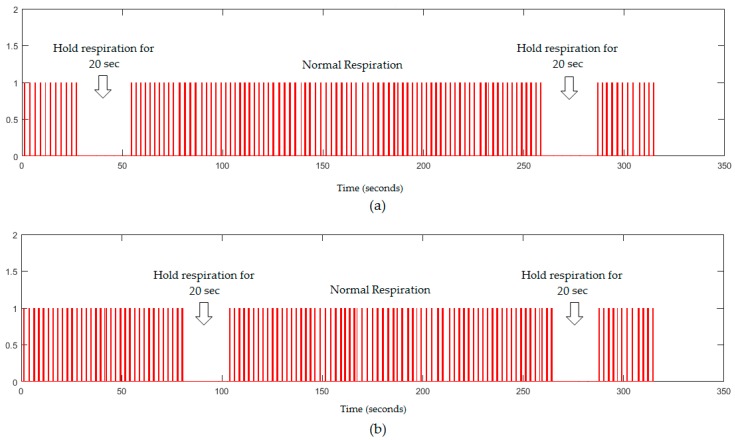
The respiratory signals (5 min) obtained from the proposed system in a dark environment, (**a**) without a blanket; (**b**) with a blanket.

From a 5 min respiratory signal shown in Figure 10, the proposed system could recognise the periods of simulated apnoea for a stopping periods of 10 s and 18 s, which represents no respiratory motion for 300 and 540 frames, respectively. The respiratory signal obtained from the proposed system in a dark environment is shown in Figure 11.

It is noted from Figure 11 that the proposed system could also recognize the periods of simulated apnoea twice for 20 s from a 5 min respiratory signal corresponding to a stopping period for 600 frames. The proposed system could recognise periods of simulated apnoea and could also send an alarm signal when breathing stopped for more than 10 s.

## 5. Discussion

In this study, a new image processing system based on the Microsoft Kinect v2 sensor was proposed to remotely monitor the cardiopulmonary signal and related abnormal events in real-time, with any given sleep posture in any environmental setting (well-lit environment and dark environment) and even when the participant is covered with a blanket. Statistical testing of results indicates that cardiopulmonary signal estimation accuracy was consistent with all tested sleeping postures and presents a high feasibility to extract cardiopulmonary information in dark environmental settings even with unclear ROI.

In the well-lit environment setting, the proposed system in the absence of the blanket showed a very good statistical agreement against the reference measurements (PCC = 0.9837, SCC = 0.9712, KCC = 0.8816, RMSE = 1.88 beats/min, and MAE = 1.64 beats/min for HR measurements, and PCC = 0.9839, SCC = 0.9681, KCC = 0.895, RMSE = 0.74 breaths/min, and MAE = 0.64 breaths/min for RR measurements). The proposed system in the presence of the blanket could also extract the cardiopulmonary signal with good agreement with the statistics (PCC = 0.9326, SCC = 0.9149, KCC = 0.7729, RMSE = 3.71 beats/min, and MAE = 3.33 beats/min for HR measurements, and PCC = 0.9145, SCC = 0.8712, KCC = 0.7389, RMSE = 1.59 breaths/min, and MAE = 1.39 breaths/min for RR measurements).

In the dark environment, the proposed system in the absence of the blanket also showed a good statistical agreement with the reference measurements (PCC = 0.9742, SCC = 0.9725, KCC = 0.8768, RMSE = 2.03 beats/min, and MAE = 1.73 beats/min for HR measurements and PCC = 0.9851, SCC = 0.9834, KCC = 0.9131, RMSE = 0.81 breaths/min, and MAE = 0.66 breaths/min for RR measurements). The proposed system in the presence of the blanket could also extract the cardiopulmonary signal with acceptable statistical agreement (PCC = 0.9341, SCC = 0.9151, KCC = 0.7907, RMSE = 3.78 beats/min, and MAE = 3.28 beats/min for HR measurements, and PCC = 0.9072, SCC = 0.8883, KCC = 0.751, RMSE = 2.1 breaths/min, and MAE = 1.63 breaths/min for RR measurements).

Regarding abnormal cardiopulmonary events, one participant was asked to do many scenarios to create similar situations to abnormal cardiopulmonary events. The experimental results with different sleep postures and different environmental settings indicated that the proposed system has the potential to detect tachycardia, bradycardia, tachypnea, bradypnea, and central apnoea even in dark environments and could send an alarm signal when heart and respiratory readings fall outside the normal range or when breathing stops.

Although the current study yields acceptable results in extracting of cardiopulmonary signal and detecting related abnormal cardiopulmonary events in many possible sleeping postures, different environmental settings, and with a covered subject, it also has some limitations. The first limitation is that the measuring range between the subject and the Kinect is limited to 4.5 m. The second limitation is that when the subject is fully covered with a blanket (with the face covered), the system will fail in tracking the ROI. There were also some system failures when the subject was lying on his abdomen and no motion could be detected.

## 6. Conclusions

This study explored the feasibility of extracting cardiopulmonary signals and detecting related abnormal events from real-time video data captured by the Microsoft Kinect v2 sensor under different environmental settings (well-lit and dark environments) and even in the presence of a blanket. The proposed system used image information from the three sensors built-in the Kinect and analysis of the changes in the abdominal-thoracic region resulting from cardiopulmonary activity using the intensity-based method and the frame-subtraction-based method. The experimental results obtained from 10 participants with different ages, sleep postures, and environmental settings indicated a strong agreement, high correlation, and acceptable error rates with respect to the reference measurements. Also, the proposed system has shown the potential to detect tachycardia, bradycardia, tachypnea, bradypnea, and central apnoea, and the option of sending an alarm signal when heart and respiratory readings fall outside the normal range or when breathing stops. The proposed system in this study would provide a comfortable and unobtrusively instrumented sleep environment for the subjects being monitored, making it potentially at the forefront of modern cardiopulmonary instrumentation technologies.

## Figures and Tables

**Table 1:** A comparison between the Microsoft Kinect v1 and v2.

Features	Kinect v1	Kinect v2
Technology used	Structured light coding technology	ToF technology
RGB sensor resolution	640 × 480, 30 fps	1920 × 1080, 30 fps
IR sensor resolution	320 × 240, 30 fps	512 × 424, 30 fps
RGB sensor Field of view	62° × 48.6°	84.1° × 53.8°
Depth sensor Field of view	57° × 43°	70° × 60°
Operative distance	0.8 m–4 m (Default) 0.4 m–3.5 m (Near)	0.5 m–4.5 m
Skeleton joints tracking	20 joints	25 joints
Number of detected subjects	2	6

**Table 2:** A normal range of physiological signs (Adapted from website: https://www.acls-pals-bls.com/algorithms/pals/). HR, heart rate; RR, respiratory rate.

Age	HR (Beats/Min)	RR (Breaths/Min)
Infant < 1 year	110–160	22–55
1–3 years	80–150	22–30
3–6 years	70–120	16–24
6–13 years	60–110	16–22
Adults	60–100	12–20

**Table 3:** Abnormal cardiac events for adult participant at two environmental settings.

States	Environmental Settings	Measured HR (Beats/Min)	Predicted HR without a Blanket	Predicted HR with a Blanket	Events
S1	Well-lit	66	68.48	69.41	Normal
S2		124	126.01	127.68	Tachycardia
S3		56	58.82	59.07	Bradycardia
S1	Dark	65	67.94	68.93	Normal
S2		130	133.05	135.14	Tachycardia
S3		56	58.13	59.62	Bradycardia

**Table 4:** Abnormal respiratory events for adult participant at two environmental settings.

States	Environmental Settings	Measured RR (Breaths/min)	Predicted RR without a Blanket	Predicted RR with a Blanket	Events
S1	Well-lit	14	14.88	15.69	Normal
S2		34	34.91	35.78	Tachypnea
S3		8	8.82	9.54	Bradypnea
S1	Dark	14	14.95	16.01	Normal
S2		33	34.05	35.14	Tachypnea
S3		9	10.04	10.83	Bradypnea
